# Experiences and Implications of the First Wave of the COVID-19 Emergency in Italy: A Social Science Perspective

**DOI:** 10.34172/ijhpm.2023.6871

**Published:** 2023-03-13

**Authors:** Serena Masino, Luisa Enria

**Affiliations:** ^1^University of Westminster, London, UK; ^2^London School of Hygiene and Tropical Medicine, London, UK

**Keywords:** COVID-19, Inequality, Public Health, Italy, Socio-Economic Impact, Social Sciences

## Abstract

**Background:** Italy was among the first countries in the world to experience the devastating consequences of the COVID-19 emergency and suffered its consequences to a devastating scale. Understanding how the country got there in spite of a relatively well-resourced public and private health system in at least part of the country, is imperative to be able to operationalise any lessons learnt for future epidemics in Italy and beyond.

**Methods:** The paper reports the findings from a research scoping exercise conducted in Italy in 2020. We conducted extensive archival research and collected 29 testimonies either in writing or as semi-structured interviews. We sampled purposively with a stratification strategy in mind, specifically aiming to gain testimonies from different social groups, classes, ages, and nature of employment. Our sample also reflects the different experiences between the Northern and Southern regions, a divide that has long been economically and politically salient in the country.

**Results:** Evidence and considerations of epidemiological nature normally guide public health responses to crises. This study supports the idea that socio-economic, cultural and political factors also affect transmission outcomes. We highlight specifically the role that socio-economic and health inequalities play in this respect, through factors such as overcrowded dwellings, lack of alternatives to in-person work, informal work set-ups, pervasive organised crime presence, poorly planned social support and communication strategies.

**Conclusion:** A socio-economic and political lens is needed in addition to an epidemiological one to fully understand the social experiences and implications of public health crises such as the COVID-19 pandemic and to devise effective response measures that are locally relevant and acceptable. Thus insights provided by multi-disciplinary task forces can render policy-making and social support interventions as well as communication strategies more effective.

## Background

**Key Messages**

** Implications for policy makers**

- National epidemic preparedness plans were not adequate with respect to both personnel and equipment supplies, but future preparedness should go beyond the simple epidemiological side of transmission.
- Socio-economic considerations other than age and pre-existing pathology should be used to determine risk profile; these should include consideration of socio-economic and health inequality.
- Where significant inequalities exist, context-specific measures should be planned to increase local relevance and effectiveness.
- In the case of Italy, geo-political and economic disparities are particularly important due to implications in terms of work informality, population density, organised crime presence, political disenfranchisement and lack of trust in institutions.

** Implications for the public**

Understanding why and how historical, political, social and economic factors converge to determine the needs of an epidemic management plan is key to helping the population reach a certain level of social cohesion around nationally mandated containment measures. By understanding why measures need to be locally relevant and appropriately communicated to be effective, mediation and social cohesion efforts can be directed towards ensuring cooperation in the identification of the most appropriate forms of social support. An open and constructive communication effort between the public, locally embedded multi-disciplinary task-forces and the government can increase transparency and policy adherence.

On February 20, 2020, a 38-year old man from Codogno, a small town in the Lombardia region of Italy, was the first in the country to officially test positive for COVID-19.^1^ Since then, Italy has taken a special place in the living history of the COVID-19 pandemic and its effects in Europe, as the first European country to experience the devastating consequences of the unfolding emergency. Images of overwhelmed hospitals, military trucks carrying coffins and of the tightly regulated lockdowns announced what was to come across the continent.^2^

 Transmission in the North of Italy had been under way, unnoticed, from at least the beginning of January 2020,^3^ or many say from the Fall of 2019.^4^ It was only in February though that evidence of the sustained spread of the disease emerged, while authorities began introducing a series of emergency lockdown measures first targeting local hotspots and eventually, from 11th March, the national territory.^4^ Draconian punitive measures were deemed necessary to halt the spread of an epidemic which was rapidly depleting the national health system’s capacity.^5^ Indeed, already on March 16, 2020, recorded cases had reached 27 980 and Italy was second only to China in terms of cumulative mortality. In the months that followed, the country’s epidemic grew further recording as of March 2021, a year on, a number of total cases and deaths per 100 000 population among the seven and third highest in the world respectively.^6,7^

 Italy’s experience with COVID-19 is an important starting point of any reconstruction of the pandemic’s effects on Europe. Understanding how the country got where it did in the Spring of 2020, in spite of a relatively well-resourced public and private health system in at least part of the country, is imperative to be able to operationalise any lessons learnt for future epidemics in Italy and beyond. However, this account is inevitably incomplete if we focus solely on transmission and epidemiological considerations. Indeed, it is increasingly recognised that, when organising outbreak responses, there is a need to also take into account the social, political and economic dimensions of epidemics.^8-12^

 This includes an understanding of how socio-cultural dynamics affect transmission, the circulation of information, and the ability to put in place effective responses. Such dynamics differ across social and geographical contexts because social policies, political economies and historical trajectories have significant effects on different countries and communities’ experiences. During the West African Ebola outbreak, for example, epidemic responders quickly realised the role of local burials practices and caring patterns in the transmission of the virus, and anthropologists supported the design of interventions that took into account social pathways of infection as well as the socio-economic impacts of restrictions.^8,13,14^ The COVID-19 pandemic has similarly given rise to a wide range of analyses highlighting the significance of social, political and economic contexts, pointing for example to the impact on transmission of different policy decisions, the relationship between marginalisation and risk of infection and the historical roots of mistrust in epidemic response measures including vaccination.^15-19^ This study contributes to this growing literature in the social science of infectious diseases, offering insights on the socio-economic, historical and political factors that contributed to the COVID-19 crisis’ trajectory in its first months in Italy. Through this analysis, we aim not only to bear witness to a collective experience of crisis, but also to note the limitations of epidemiological approaches that do not take into account how localised political economies structure the experiences and consequences of epidemics and their associated interventions.

 In this study, we use personal testimonies and semi-structured interviews collected across Italy in the months between February and October 2020 as a starting point to highlight social, economic and political factors that we deem relevant for understanding the impact of the COVID-19 pandemic during Italy’s first wave. We contextualise these testimonies with an analysis of the pandemic’s trajectory, drawing on newspaper articles and a review of official reports. The aim of the article is threefold: (*i*) to bear witness to the individual and collective experiences in Italy’s first wave of the pandemic, (*ii*) to support an understanding of the COVID-19 pandemic that takes into account Italy’s particular socio-political context, and (*iii*) to identify recommendations for future interventions. In what follows, we first present our data and methodology, we then move onto the findings which are divided into thematic areas, each of these is discussed in order to derive social and health policy implications.

## Methods

 The data used in this article were collected either in the form of semi-structured interviews or written testimonies. We utilised rapid evidence assessment qualitative methodologies, which have been increasingly applied in the context of health emergencies.^20^ These methodologies include working from interview notes rather than transcription, rapid approaches to analysis and the use of multiple rapid methods. During COVID-19, these methods have also by necessity included the use of social media, email, WhatsApp and phone calls to quickly collect testimonies. This was particularly important for our study in March 2020, as we began collecting our testimonies with a view to share the knowledge with international organisations as the pandemic unfolded and their rapid decision-making needed an informed basis of field data and evidence. This article builds on that initial work and an informal internal report we produced. Despite the use of this rapid methodology dictated by the condition of emergency, we sampled purposively with a stratification strategy in mind, specifically aiming to gain testimonies from different social groups, classes, ages, and nature of employment. Our sample also reflects the different experiences between Northern and Southern regions, a divide that has long been economically and politically salient in the country and that, during the first wave of the pandemic, accounted for marked differences in COVID-19 caseloads and responses. More specifically, we included respondents from seven Regions, two in the South (Campania and Calabria) and five in the North (Liguria, Lombardia, Toscana, Emilia Romagna, and Friuli Venezia Giulia). We carried out a total of 13 interviews in southern regions (11 in Campania and 2 in Calabria) and collected 16 testimonies from northern regions (7 from Liguria, 6 from Lombardia, and 1 each from the remaining three Regions). The sample was determined by access; with those targeted by requests for interviews or testimonies determined by gatekeeper dynamics and subsequently via snowballing. Those who agreed to participate in interviews and/or provide were included in the final sample.

 The interview guides contained questions on respondents’ demographics, personal experience of the pandemic, impact suffered through their employment and living conditions, their opinion of the institutional response and their expectations for the future, including what type of support they had access to or were likely to keep receiving. The semi-structured interviews were collected among 13 respondents, with 15 having been initially approached. Written testimonies were solicited by email, with a short blurb circulated on social media and by email, requesting for the email to be shared with others. The blurb explained our role as researchers and asked for contributions to help us write a report on social experiences affecting people in areas hit by coronavirus, and that we were particularly interested in reflections on personal experiences from healthcare workers (HCWs), socio-economic impacts of lockdown and any challenges associated with COVID-19 regulations. Sixteen respondents sent their written testimonies. We followed an ethical protocol approved by the researchers’ institutions and stored the data safely on University drives after having thoroughly anonymized it. In order to contextualise the data that reflects personal and collective experiences, our thematic analysis of the testimonies and interviews was complemented by extensive archival research. More specifically, we conducted a review of newspaper articles from leading national newspapers and local news as well as official reports from research institutes or organisations.

## Results

###  Effects of COVID-19 on the Health System 

####  Differences in Timing of Transmission and Reactions Across the National Territory

 The rapid onset of the COVID-19 epidemic in Italy followed a delay in recognising and detecting the spread of the virus in January 2020. This allowed the contagion to progress unchecked for weeks and to grow exponentially in Lombardia.^4^ As work-related movements are frequent, the disease was quick to spread to all other regions within a matter of weeks. However, while Lombardia, and more in general the North of Italy, have a well-resourced health system and one among the best in the world, the same cannot be said of the rest of the country, where the state of the healthcare system often reflects the decades of divided dual-speed growth experienced by the country.^21^

 Against this background, the potential for widespread transmission beyond Northern regions was particularly worrying and concerns intensified when the announcement that a lockdown would be enforced in Lombardia and some surrounding areas was leaked to newspapers. The leaked information, in fact, gave thousands of residents, who lived in Northern regions for study or work reasons but whose families were from the South, the time to board trains and buses, literally overnight, directed towards the South.^22^ Unsurprisingly, many of them were knowingly or unknowingly infected prior to or while travelling. In spite of this, however, many self-isolated upon arrival spontaneously, something which contributed to the first wave of the pandemic broadly sparing Southern Regions the crisis that engulfed the health systems in the North. Indeed, the time lapse before the initial realisation the virus was spreading in the North and fears raised by reverse migration seem to have allowed the Southern regions to prepare themselves and lock down pre-emptively, something which differed markedly with the experience of the following second wave in the autumn of 2020, as we will discuss below.

####  Under-Resourced Hospitals and Healthcare Workers

 As beds for the hospitalised were not freeing up at pace with new case numbers in Lombardia, private and public bed spaces in intensive care units (ICUs) rapidly filled up as of mid-March 2020 already. Many other regions in Italy only had a fraction of the ICU availability of Lombardia, particularly in the South. The risk was therefore that, if the spread of the disease went unchecked, entire regions could have been completely unable to provide ventilation, resuscitation, and on-going care for critical COVID-19 patients, as well as emergency care for other patients with significant co-morbidities. Such possibility generated controversies around the potential necessity of triage to prioritise intensive care for younger patients or those more likely to recover.^23^ According to a number of reports, such difficult decisions had already been implemented in the most affected areas of the North.^24,25^

 The suspension of funerals as part of the measures to stem transmission meant that morgues were becoming full. In the province of Bergamo, a particularly badly affected area, the death of a mayor was accompanied by the distressing news that burials in Val Seriana were happening “every half hour.”^26^ A later judicial investigation would find public health administrative directors in the province potentially liable for gross misconduct and failure to implement protocols mandated by the World Health Organization (WHO).^27^

 Another risk factor was associated with the nationwide shortage of HCWs and of their personal protective equipment (PPE)^28^; shortages which were compounded by a delay on the part of the European Union to intervene in support of the Italian health system.^29,30^ Alarmingly, in our discussions with HCWs in Campania and Calabria, it became clear that that family doctors – that is, the primary healthcare providers – were lacking PPE throughout the crucial initial weeks of the pandemic. As Italian family doctors are considered self-employed professionals, there was a delay, or unwillingness according to some, to step up the provision of PPE, while supplies in shops and pharmacies very rapidly depleted:

 “*We received one single PPE kit for all doctors in the municipal district to share (…) this is because we are considered as self-employed professionals. We tried to buy our own PPE but it is impossible, it has run out virtually everywhere. (…) The government will send them out only when it is too late, I needed protection now and earlier over the past few weeks”* [General Practitioner, Calabria].

 Combined with the fact that most HCWs were initially only encouraged to wear a mask when coming in contact with symptomatic patients, this meant that family doctors and many other categories of HCWs carried out their duties throughout the critical initial phases of the pandemic without any adequate protection. This was particularly concerning as the evidence of widespread asymptomatic transmission had already started to emerge.^31-33^

 In hospitals, nurses and doctors were working long shifts and foregoing their annual leave; yet staff shortages were common due to illness or self-isolation, until a controversial governmental decision on ninth March (Decree n.14 9/3/2020), which asked all quarantined HCWs to resume their duties immediately if they did not display symptoms, even if they had had contacts with potentially infectious patients. While such a decree was meant to stem the loss of further precious personnel resources, it unsurprisingly generated bitter protest among HCWs, who were concerned both about their own health and about the risks that hospitals could rapidly transform into clusters of nosocomial transmission.^34^ Indeed, Erdem and Lucey reported that Italy was among the countries with the highest HCW mortality rate by population, with the estimate standing at 0.35 per 100 000.^34^

 In a similar way, shortages of testing kits were the reason why it was established by the Italian authorities that only those admitted as inpatients could get a COVID-19 test. The decision raised concerns that transmission may be further fuelled by undetected infection, precisely at a time when the WHO changed its guidelines to encourage countries to step up testing efforts in an attempt to stem transmission carried by asymptomatic and mild cases.^4,35^

####  Co-morbidity and Effects on Non-COVID Health Services

 Outbreak case counts often obscure the broader effects on those suffering from other diseases. As Regions across Italy began suspending all planned procedures and accident & medical clinics of non-urgent nature, only the service of haemato-oncologic patients and other cardiovascular emergencies was guaranteed to free up resources for the COVID-19 emergency. In addition to this, even those with significant and urgent pathologies were often reluctant to attend their medical appointments for fear of contagion. Thus, another main concern beyond the epidemic of COVID-19 *per se *became co-morbidity. Italy’s ageing population, with 22% of the population being over 65,^36^ made the threat from co-morbidities even more serious.^37^

###  Inequality, Employment and the Economy

####  Living Conditions and Inequality 

 An additional element to consider is that medical conditions, especially those of chronic nature, may be more prevalent among the low-income sections of the population given their impaired or diminished access to adequate healthcare.^38^ This, combined with living and working conditions that do not allow for effective social distancing make this part of the population particularly vulnerable. For example, living conditions in some of the urban areas in Southern Italy where our respondents were based, made it almost impossible for social distancing and containment measures mandated by central and regional governments to work. These areas are mostly home to low-income households and have very high levels of population density, with the poorest often co-habiting in a one-room open space.

####  Employment and Inequality 

 In 2020, Italy’s gross domestic product growth rate dropped to -8.8%.^39^ The entrepreneurial community in the industrialised North pushed for continued economic activity, a message that was shared and reinforced by the main Italian Industry Confederation, *Confindustria.*^4^ While public and private employers made efforts to facilitate home-working and a government-led ‘digital solidarity’ initiative made available online collaborative working platforms (known in Italian as ‘*smart working*’); the coordinator of a private sector industrial consortium in the northern region of Lombardia noted:

 “…*those who work on the factory floor can’t do ‘smart working’” *[Private Industry Consortium Coordinator, Lombardia].

 In some cases, these tensions resulted in strikes and contestations.^40,41^ The ability and willingness of different companies to comply with newly mandated protective measures in order to stay open was far from uniform and a former opposition party leader continued to report complaints from workers, who felt they were being asked to keep on working while not being adequately protected.^42^

####  Self-employment

 Certain business sectors and worker categories proved to be particularly vulnerable. Italy’s entertainment and tourism sectors – key sectors for the nation’s economy – unsurprisingly took a significant hit, affecting with it from waiters to chefs, from tour guides to owners of bars and restaurants. A category that was also significantly affected was that of freelancers. The self-employed landscape in Italy separates between professionals such as lawyers or accountants – who receive protection from dedicated professional bodies – and freelancers, who work on a largely more causal and less formally protected way.

 There was a gap of weeks before the government announced any support measures, which left many without a source of income but still liable for bills and rent. A series of wide-ranging measures were finally announced in mid-March including the provision of sick pay for people in quarantine, the suspension of taxes and mortgage repayments, the reduction of utility bills for the whole of 2020 and a transfer of 600 euros to the self-employed.^43^

####  Unemployment and Informal Employment 

 Although nation-wide measures were put in place in the summer 2020 to forbid individual firing and redundancies,^44^ many lost their jobs before that decree came into place due to reduced overall economic activity, bankruptcies, or because they worked informally, as a hairdresser in Campania explained:

 “*I was on probation when COVID-19 struck, they wanted to hire me on a formal contract, but this fell through as they cannot afford to pay for a share of my furlough”* [Hairdresser, Campania].

 More complex narratives also emerged where precarious employment compounded issues of co-morbidity and lack of access to healthcare, as in the following testimony by a pastry-maker:

 “*I was supposed to undergo surgery on 16th March, but the lockdown meant it got cancelled. I wasn’t able to undergo surgery until the following September. So, for now, I still have not been able to get a job (…) which meant I ended up in long-term unemployment” *[Pastry Chef, Campania].

 As in other parts of Europe, work flexibilisation pressures have risen steadily in Italy in the last thirty years,^45,46^ following laws such as the Biagi Law of 2003 whose impact on increasing precariousness for younger generations is still the subject of debate.^47^ The result is that today, in Italy, a significant proportion of people work in the informal or ‘unobserved’ economy, this includes both Italian and migrant workers. Italy’s main statistics agency (ISTAT, Istituto nazionale di statistica) calculated in 2016 that the ‘unobserved economy,’ made up of underreported illegal activities like drug trafficking and prostitution and irregular work, amounted to around 12.4% of gross domestic product.^48^

 A number of social and income support funds exists to supplement the incomes of those who lose their jobs and/or are unemployed,^49-51^ but they are tied to requirements of citizenship and residence status. Informality is common in Italy both in the rental and job markets,^52,53^ this means that many, citizens and undocumented migrants alike, did not qualify for such social assistance upon losing their jobs as a result of the pandemic. Significant differences are likely to compound over time along the regional dimension, given that Southern regions experience more pervasiveness of informality and that the vulnerability deriving from informal work is higher in these Regions.^30^

 Various initiatives advanced requests for an alternative form of income supplementation that anyone struggling to meet their basic needs due to COVID-19 could apply for, regardless of employment type, citizenship, or residence status. Unfortunately, while it emerged from our interviews that a number of private and public grassroot initiatives were organised including the establishment of food banks, no concrete steps were taken to establish a government-run income supplementation scheme with universal coverage. In May a time-limited emergency fund was approved for households suffering economic difficulties as a result of COVID-19; once again, however, anyone who could not prove their residence status was excluded from the scheme.^54^

 This fuelled widespread complaints that national support measures had been laid out in a way that favoured Northern regions, while the specific reality of the South had been overlooked, as denounced in the following testimony by a personal trainer in Campania:

 “*If the government wants to keep people home due to the pandemic, better forms of support are needed, taking into account differences in territories (…) *[in the South]* many work informally, if we want logical rules that can be respected by all, they need to be created by politicians who know the local reality” *[Personal Trainer, Campania].

 The narrative became particularly powerful as it strengthened an already existing record of exclusion and discrimination between different areas of the country.

####  The Reality of Migrants in Italy

 Similar concerns were raised in relation to the migrant population, which was estimated to be in excess of half a million people in Italy in 2020.^55^ A local NGO coordinator from Campania, explained that, aside from health-related matters, socio-economic concerns were found to be most pressing due to the widespread informality in migrants’ employment arrangements. In addition, employers’ fears of transmission further reduced work opportunities, be it casual odd jobs or permanent jobs as home carers for the elderly.

 “*We receive hundreds of daily calls asking for help (…) as people now think twice before employing someone in their homes, and for many migrants who worked informally it is impossible to justify going out during the lockdown without a contract” *[Local NGO Coordinator 1, Campania].

 The main concern among volunteers and social workers was that many had being going without food for days and would have been at serious survival risk without the network of support that was swiftly established at the grassroot level.

###  Social Care

####  The Elderly

 Significant anxiety was associated with the repercussions on elderly relatives and their ongoing care needs, as it is apparent in the testimony below by a resident of a town in Liguria:

 “*The difficulties are not only felt from an economic but also from a psychological point of view. In spite of having so much free time now that we are in lockdown, I cannot concentrate, let alone relax, as we are forced into a permanent state of anxiety. The media bombard us with distressing messages, fake news are raging on social media and we hear from the windows the constant sound of police cars and their speakers blasting orders to stay home. All the while, I keep thinking of my parents who live alone as they are separated and my grandmother who is in her 90s and is feeling so scared that she hasn’t let me in in a week and she doesn’t even leave the house to dispose of garbage” *[Gym instructor, Liguria].

 Many of the elderly who lived on their own were indeed left frightened and entirely isolated, as they had to rely on shop-owners to periodically bring some groceries to their house. As mentioned above, Italy has an ageing population among the largest in the world.^4^ A ‘familialistic’ or informal care orientation implies that family members take the primary role and are the main recipient of welfare support in relation to the care of the elderly,^56,57^ who often either live at home with younger relatives or rely on their regular visits to get support with first necessities. In turn, grandparents are often the primary providers of childcare. These circumstances are, however, associated to significant implications in terms of transmission dynamics, given that COVID-19 has markedly different risk profiles depending on age.

###  Institutional Trust, Communication and the Social Response

####  Trust in the Institutional Response

 In Italy, trust in institutions has historically been low. A Eurobarometer survey in 2018 showed for example that 66% of the population tended not to trust the government.^58^ Volatile governments, regular corruption accusations and significant political divisions have contributed to this in recent years.

 A context of mistrust is relevant for understanding initial concerns around adherence to government regulations. In the first weeks of the emergency, there were for example reports of people escaping quarantines from Northern locked down areas. Rumours also circulated widely on traditional and social media that mischaracterised and underplayed the epidemic comparing it to an ordinary flu; or, in other cases, spread false information leading to panic. While many, especially among the wealthier classes, worried about the broader consequences resulting from the reluctance to comply with regulations on the part of the youth and all those who lacked adequate information or sense of community belonging,^4^ as conveyed by the following response by a physician in Campania:

 “*The government should frighten [the youth]. We should not make up lies but people will start to comply only when they realise that full-capacity ICUs will soon be reality” *[Paediatrician, Campania].

####  Communication Challenges

 A significant challenge for building trust in the national response during the first wave was particularly around the perception of confused, or even contradictory, communication. This, as one of our interlocutors, a communications specialist based in Tuscany, put it, was both in terms of ‘vertical’ communication between different tiers of the institutional hierarchy and external communication from the government to the public. For example, tensions around COVID-19 testing meant municipal health centres were receiving contradictory protocols around whether to do ‘blanket testing’ or test only those who were symptomatic. A second example is the leak of the ordinance relating to the lockdown of a number of northern Regions, as described earlier, due to which the decree was published in full in a major national newspaper before the government announced it.

 Some of our testimonies also focused on the perception of contradictions in the ordinances not necessarily in terms of lack of information but rather due to too much, at times contradictory, information. Indeed, disagreements among experts and authorities played out quite publicly in Italy, with social media and TV shows rife with debates but a dearth of explanation about the rationales behind specific response measures.

 Other information campaigns and social mobilisation efforts have had more success, including the viral hashtag #iorestoacasa (‘*I am staying home’*),^59^ the engagement of celebrities in the sharing of public health messaging and community-led musical ‘flash-mobs’ from people’s windows and balconies to strengthen a sense of national solidarity during the lock-down.^60,61^

####  Social Cohesion 

 Multiple counterbalancing examples of solidarity and social cohesion also emerged as well as of adherence to the measures from across the national territory. A number of testimonies have in fact pointed out to a sense of responsibility shared by local communities in following the government’s lockdown regulations; with business owners leading the effort by closing down their premises or, where needed, installing social distancing aids and signals. It also emerged from our interviews that various corporate, including through large bank donations, and grassroot food bank initiatives were established to provide help to all those who were not able to access formal government help due to their informal working arrangements, including undocumented migrants.

####  Organised Crime

 Questions of institutional trust and social cohesion cannot be fully understood without considering their link to the long-standing presence of organised crime on the Italian territory. Such endemic presence is, in fact, typically associated with both strained relationships with authorities and low sense of belonging to any national identity. This results in low levels of compliance with government-mandated provisions in certain sections of the population which have mostly experienced a recurrent absence of the State from the socio-economic relations in their lives. Against this backdrop, reports of organised crime interventions to coordinate the social support response during the first wave of the epidemic in the South of Italy were seen as unsurprising by many, as the coordinator of a migrant centre in Campania explains:

 “*Organised crime establishes itself in the empty spaces left by the State. People have been asking for income support for weeks now, and they have been denouncing the economic catastrophe they are heading towards in the South, where 40% of the population either does not have a job or has one which is precarious or informal” *[Local NGO coordinator 2, Campania].

 A more far-reaching risk was identified in the expansion of organised crime’s realm of influence, particularly through money lending and money laundering, as argued by an expert on organised crime in the South of Italy^62^:

 “*The data shows that street crime declined sharply but illegal money lending increased by 10%, and that’s only the part reported to the police. This is an unprecedented opportunity for money laundering which not even the 2008 financial crisis provided. For example, if a hotel owner in Napoli is struggling, organised crime will offer a very good price to buy off the hotel. It is a tried and tested mechanism which, through the acquisition of local businesses, offers a way to clean the profits of drug trafficking” *[Analyst, Campania].

## Discussion

 The research for this project was conducted using rapid methodologies to document an unfolding crisis. Consequently, the study has several limitations, including a limited sample that cannot be regarded as representative of much broader society-wise points of view. In this article, we have also selected the topics that came out most coherently from our data, leaving out other important themes (eg, gendered experiences of lockdown, including disproportionate workloads and heightened domestic violence)^4^ that emerged from the data less cohesively.

 Our findings explored a number of socio-economic and public health dimensions that accompanied the first few months of the rapidly spreading first wave of COVID-19, that is, at a time when the country had been caught unprepared and the government had not yet announced relief measures. These findings therefore reflect a transitory phase, but one that was crucial in determining a host of socio-economic tensions and an initial climate of mistrust in the public health response that had long-lasting consequences. More specifically, we described how, after transmission went unnoticed for at least the whole of January 2020,^3^ the Italian healthcare system rapidly became overwhelmed in various parts of the North of the country. Scarce essential PPE in the critical initial months of the pandemic accompanied HCWs shortages as personnel got ill or quarantined, against a backdrop of spiralling death numbers and rapidly filling morgues.

 A number of policy-relevant points emerge: the first is that governance oversight and administrative delays meant family doctors lacked PPE throughout the first few weeks of the outbreak, something which exposed them to unnecessary danger and aggravated community transmission.^4^ The self-employed status of family doctors was thought to be relevant in explaining such delay in the distribution of PPE. Future outbreaks would need to take into account the transmission implications of leaving unprotected the health-system tier that more closely comes in contact with the community.

 Secondly, a number of early decisions were taken with respect to access to tests and PPE, with tests reserved for inpatients, while self-isolating HCWs were recalled to work if asymptomatic. This was in contrast with both the emerging evidence of widespread asymptomatic transmission^31-33^ and the newly issued guidelines by the WHO inviting countries to step up testing efforts in an attempt to stem asymptomatic transmission. While widespread supply shortages were at the origin of such decisions, they resulted in heightened risk of spread within the community and the danger that hospitals could transform into clusters of nosocomial transmission.^31^ Indeed, an inquest into the public health system crisis in the province of Bergamo, the worst affected hotspot in the North, revealed that hospital transmission played a considerable role in the initial phases of the epidemic, at a time when pandemic preparedness plans were outdated by over ten years and had not been timely triggered in spite of formal warnings by the WHO.^4,27^

 Thirdly, the Italian experience of the COVID-19 pandemic calls into question the common understanding of vulnerable categories from the point of view of transmission. These categories are normally defined as those with pre-existing pathologies and the elderly, but we argue that location-specific transmission dynamics should guide the identification of socially vulnerable groups, in particular to include in the context of Italy those living in overcrowded conditions such as in low-income neighbourhoods or migrant centres, who face specific challenges in relation to self-isolation. In this respect, the Community Care Centres model established in previous epidemics^63^ provides food for thought for alternative self-isolation and treatment arrangements.

 As one of the most at-risk groups from the pandemic, the protection of the elderly proved particularly challenging in the context of the Italian familialistic long-term care orientation. Indeed, the pandemic has greatly increased the need for care, and especially that of the unpaid type^64^ this underscores a parallel emerging need for appropriate guidelines for households engaged in the provision of such care. In particular, as the elderly normally receive support from younger relatives and look after children in the household, a delicate balance needs to be achieved between minimising the psychosocial impacts of isolation and the transmission implications of inter-generational mixing. An important additional point in relation to the analysis of transmission dynamics is the comparative dimension between Northern and Southern Italy and the emerging importance of appropriately timed lockdowns. In fact, evidence emerged that during the first wave, the entrepreneurial community in the industrialised North pushed for continued economic activity, a message that was shared and reinforced by the main Italian Industry Confederation, *Confindustria.*^4^ To the contrary, a different production landscape and attitude of authorities towards it, coupled with the time lapse after the identification of widespread transmission in the North, allowed Southern regions enough time to lock down pre-emptively. This was no longer an option in the Autumn of 2020 when community transmission was already much more sustained everywhere across the national territory and the dire consequence of the first lockdown had pushed many in the South to resume their economic activities and occupations.

 Indeed, even though the first wave of the epidemic fell short of causing in southern Regions levels of crisis similar to the North, what hit them most were the socio-economic consequences of the lockdown. The Italian government passed unprecedented legislation to support workers, including those working on precarious contracts and the self-employed. However, on the one hand, this did not happen straight away and some categories of workers were left out, such as factory workers — who were still required to go to work — and irregular workers. For the latter, in particular, the absence of a contract meant either that they could not justify leaving home under the very strict lockdown monitoring by the police or that if work stopped they could not access furlough and social support schemes. Similar dynamics applied to Italian and migrant informal workers alike; yet, in spite of the importance that the informal sector covers in the Italian economy, no concrete institutional steps were taken to establish an income supplementation scheme with universal coverage that effectively took into account the reality of a large chunk of the vulnerable population.

 As it has been shown that the population in Southern regions is more vulnerable to the precariousness created by informal work,^53^ complaints were raised that the institutional response to the pandemic was skewed towards the interest of the North. This reflects historical patterns of structural regional inequality^65,66^ and exacerbates politically-relevant perceptions of it. In this respect, a risk was also identified in the danger of organised crime’s acquisitions of struggling businesses in the wake of the pandemic, as part of widespread exploitation and money laundering operations in the South. However, an effort to stem further infiltration in the productive socio-economic landscape as a consequence of the COVID-19 induced economic crisis appeared missing.

 Perceptions of a North-South divide, of structural inequality, and of the State’s absence behind organised crime’s expansion all combine to determine the historically low levels of institutional trust in Italy. In this respect, recent epidemics, including the Ebola outbreaks in West Africa, have highlighted the central role played by trust in the challenges related to the implementation of outbreak response measures.^67-69^ While strong community-led mobilisation inspired by solidarity and numerous examples of social cohesion around government-mandated measures appeared in our testimonies, confusing institutional communication still partially undermined trust in the public response to the epidemic, especially in the early stages. Specifically, ineffective vertical communication among institutional levels took place alongside contradictory information circulated externally to the public through a variety of channels, including social media. A lack of concerted effort to harmonise public messages resulted in too much, too vague, information that was difficult to absorb and rationalise.

 Prior epidemics have shown the importance of clear and coherent community engagement and risk communication in supporting campaigns to increase the acceptability of outbreak response.^70^ In particular, response task forces should consult latest guidance on meaningful community engagement to develop initiatives relevant to the Italian context and that encourage participation not only at the national but also at the local level, by appropriately reflecting and representing local concerns and understandings.^71^ This is more easily achieved when trusted social networks, for example local social movements, football associations, or parent groups, are identified and engaged. In the context of Italy, it might also mean developing regionally specific communication campaigns to divulge national messages in ways that are locally relevant. Finally, effort should be placed on the identification of latent or explicit social conflicts and marginalised groups so as to develop more targeted messaging.

 The importance of all such socio-political dimensions, beyond epidemiological consideration, underscores the need for ensuring that the right stakeholders are sitting at the table when the institutional response to public health emergencies is devised. More specifically, task forces should include competencies from different sectors of society; beyond political representatives. This of course includes scientific and medical professionals, but it should also encompass local community representatives, cultural mediators, communication experts, and social scientists to ensure relevance and viability in local contexts.

 Bearing in mind the limitations described above and the time-limited nature of the analysis we undertook in this study; further research avenues should concentrate, first of all and in the context of Italy, on comparing if and how the policy response and the socio-economic outcomes differed in the subsequent phases of the epidemic. Furthermore, more work aimed at generating comparative perspectives between instances of successful local participatory governance of epidemics in contexts beyond Italy is needed.

## Conclusion

 This analysis draws evidence from Italy which was the first country in Europe and one of the first in the world to face the COVID-19 pandemic. Italy was also among the worst hit countries in the world in the first phase of the pandemic. The aim was to document social experiences and perspectives through personal testimony as the pandemic was unfolding. In so doing, we have highlighted the importance of considering a host of socio-economic, cultural, and political factors that affect transmission outcomes alongside epidemiological factors. The analysis has sought to offer complementary tools and perspectives from the social sciences realm that can render policy-making more effective in future public health crises in and beyond Italy. We identified a number of policy-relevant dimensions connected to the decisions to delay the protection of family doctors and neglect the emerging evidence on asymptomatic transmission in the early phases of the outbreak response. We further highlighted that socio-economic considerations other than age and pre-existing pathology should be used to determine risk profile. Specifically, where significant inequality exists, context-specific measures should be planned. In the case of Italy, this should particularly take into account geo-political and economic disparities as well as the implications of widespread informality, high population density, and the living conditions among the poorest in relation to self-isolation. Finally, we shed light on how ineffective communication strategies can strengthen pre-existing low levels of trust in institutions and thus hamper the effectiveness of outbreak response measures. In this respect, we suggested future health policy-making should endeavour to review lessons learnt from prior epidemics, as a means to improving the planning of vertical and external communication and its relevance for local contexts so as to increase uptake. In particular, including social scientists alongside medical professionals and politicians in the relevant task forces can help achieve the latter.

## Ethical issues

 The study received ethical clearance from both the University of Bath and the University of Westminster.

## Competing interests

 Authors declare that they have no competing interests.

## Authors’ contributions

 SM: literature review, data collection, analysis, and writing up. LE: literature review, data collection, and analysis.
